# Supplementary material for: Repeat expansion in a Fragile X model is independent of double strand break repair mediated by Pol θ, Rad52, Rad54l or Rad54b
Source: bioRxiv. 2024 Nov 6:2024.11.05.621911. Preprint. [Version 1] doi: 10.1101/2024.11.05.621911 (PMC11580960; doi:10.1101/2024.11.05.621911)
Supplement: Supplement 1 [file NIHPP2024.11.05.621911v1-supplement-1.pdf]

bioRxiv preprint doi: https://doi.org/10.1101/2024.11.05.621911; this version posted November 6, 2024. The copyright holder for this preprint (which was not certified by peer review) is the author/funder. This article is a US Government work. It is not subject to copyright under 17 USC 105 and is also made available for use under a CC0 license.

Supplemental Material

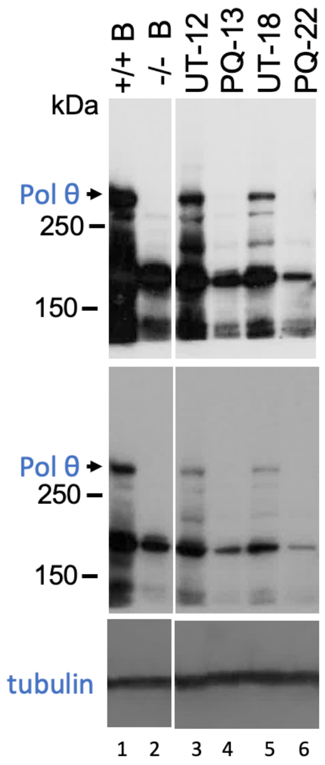

Supplemental Figure 1. Confirmation of *Polq* gene knockout in mouse cell lines. An uncropped immunoblot of extracts from the indicated cell lines, using an antibody recognizing mouse Polθ. Extracts were prepared from B cells from *Polq*$^{+/+}$ mice (lane 1); B cells from *Polq*$^{-/-}$ mice (lane 2); *Polq*$^{+/+}$ mouse cell line WT-12 (lane 3); *Polq*$^{-/-}$ mouse cell line PQ-13 (lane 4); *Polq*$^{+/+}$ mouse cell line WT-18 (lane 5); *Polq*$^{+/+}$ mouse cell line PQ-22 (lane 6). The top two panels show darker and lighter exposures of the same immunoblot. Intervening lanes containing irrelevant samples were excised from the immunoblot as indicated. The 50 kDa region of the gel was cut away and immunoblotted separately with an antibody against alpha tubulin, shown in the bottom panel.

Supplemental Methods

bioRxiv preprint doi: https://doi.org/10.1101/2024.11.05.621911; this version posted November 6, 2024. The copyright holder for this preprint (which was not certified by peer review) is the author/funder. This article is a US Government work. It is not subject to copyright under 17 USC 105 and is also made available for use under a CC0 license.

Extracts were prepared by lysis of pellets from 5 x 10^6 cells. Cells were resuspended in buffer containing SDS, boiled for 10 min, sonicated, and insoluble material was removed by centrifugation. Samples (15 µL for B cells, 10 µL for other samples) were loaded on a 3-8% gradient Criterion™ XT Tris-acetate protein gel and run with XT running buffer at 75 V for 180 min. Markers were All Blue Precision Plus Protein standards (Bio-Rad). Following transfer and drying of the membrane, immunoblotting used mouse monoclonal antibody against Pol θ. This antibody (153-5-1) was raised against a fragment of Pol θ QL [46]. Purified antibody (2.4 mg/mL) was used at 1:500 dilution in blocking buffer. Following washing, secondary goat anti-mouse HRP antibody was used at 1:10,000 dilution in 5% non-fat dried milk (CST) in TBS-T solution. The film was developed with Clarity plus reagent and exposed to x-ray film. A separate identical gel was loaded and stained with Revert total protein stain and imaged on a Li-Cor to confirm equal staining.

Preparation of mouse B-cell extracts.

Polq$^{-/-}$ mice, originally derived by Shima *et al.* [56], were obtained from Jackson Laboratories as described and maintained on a C57BL/6J background [57]. Naïve mouse B cells were isolated from spleens, negatively sorted with anti-CD43 beads, and cultured with lipopolysaccharide and interleukin-4 as described [58]. Extracts were prepared after 72 h culture.
